# Epicardial and pericardial adipose tissue: anatomy, physiology, imaging, segmentation, and treatment effects

**DOI:** 10.1093/bjr/tqaf223

**Published:** 2025-09-13

**Authors:** Tristan T Demmert, Konstantin Klambauer, Lukas J Moser, Victor Mergen, Matthias Eberhard, Hatem Alkadhi

**Affiliations:** Diagnostic and Interventional Radiology, University Hospital Zurich, University of Zurich, Zurich, 8091, Switzerland; Diagnostic and Interventional Radiology, University Hospital Zurich, University of Zurich, Zurich, 8091, Switzerland; Diagnostic and Interventional Radiology, University Hospital Zurich, University of Zurich, Zurich, 8091, Switzerland; Diagnostic and Interventional Radiology, University Hospital Zurich, University of Zurich, Zurich, 8091, Switzerland; Diagnostic and Interventional Radiology, University Hospital Zurich, University of Zurich, Zurich, 8091, Switzerland; Diagnostic and Interventional Radiology, University Hospital Zurich, University of Zurich, Zurich, 8091, Switzerland

**Keywords:** epicardial adipose tissue, pericardial adipose tissue, cardiac imaging, computed tomography, cardiovascular risk, echocardiography, deep learning

## Abstract

Epicardial adipose tissue (EAT) and pericardial adipose tissue (PAT) are increasingly recognized as distinct fat depots with implications for cardiovascular disease. This review discusses their anatomical and physiological characteristics, as well as their pathophysiological roles. EAT, in direct contact with the myocardium, exerts local inflammatory and metabolic effects on the heart, while PAT influences cardiovascular health rather systemically. We sought to discuss the currently used imaging modalities to assess these fat compartments—CT, MRI, and echocardiography—emphasizing their advantages, limitations, and the urgent need for standardization for both scanning and image reconstruction. Advances in image segmentation, particularly deep learning-based approaches, have improved the accuracy and reproducibility of EAT and PAT quantification. This review also explores the role of EAT and PAT as risk factors for cardiovascular outcomes, summarizing conflicting evidence across studies. Finally, we summarize the effects of medical therapy and lifestyle interventions on reducing EAT volume. Understanding and accurately quantifying EAT and PAT is essential for cardiovascular risk stratification and may open new pathways for therapeutic interventions.

## Introduction

Epicardial adipose tissue (EAT) and pericardial adipose tissue (PAT) are increasingly recognized as key contributors to cardiovascular disease. This review aims to provide a comprehensive discussion on the physiological and pathological role of EAT and PAT, focusing on imaging techniques for their evaluation and the emerging role of machine learning in their quantification. Potential treatment effects on EAT and PAT volume, as assessed with imaging, are discussed.

## Anatomy

The term epicardial fat was first introduced in the early 20th century to describe the adipose tissue located between the visceral layer of the pericardium and the myocardium.^1^ The pericardium serves as the anatomical boundary between the EAT and PAT and appears on imaging as a thin layer (with a thickness of 1.0-4.0 mm), which is sometimes difficult to discern.^2^ The fat outside the pericardium lacks terminological consistency and shows considerable variability across the literature. Previous studies denominate it intrathoracic fat,^3^ paracardial fat,^4^ mediastinal fat,^5^ or pericardial fat.^6^ Zaleska-Kociecka et al^7^ and Kim et al^8^ described pericardial fat as a separated depot of fat in-between the visceral and parietal pericardium, and Rosito et al^6^ defined pericardial fat as any adipose tissue located within the pericardial sac.

In this review, we defined the thoracic fat compartments as follows and consistent with Bertaso et al^9^ (Figure 1). Epicardial fat refers to visceral intrapericardial fat contiguous with the myocardial surface. Pericardial fat is defined as fat outside the parietal pericardium, acknowledging that there is no adipose tissue between the pericardial layers.^10^

**Figure 1.**
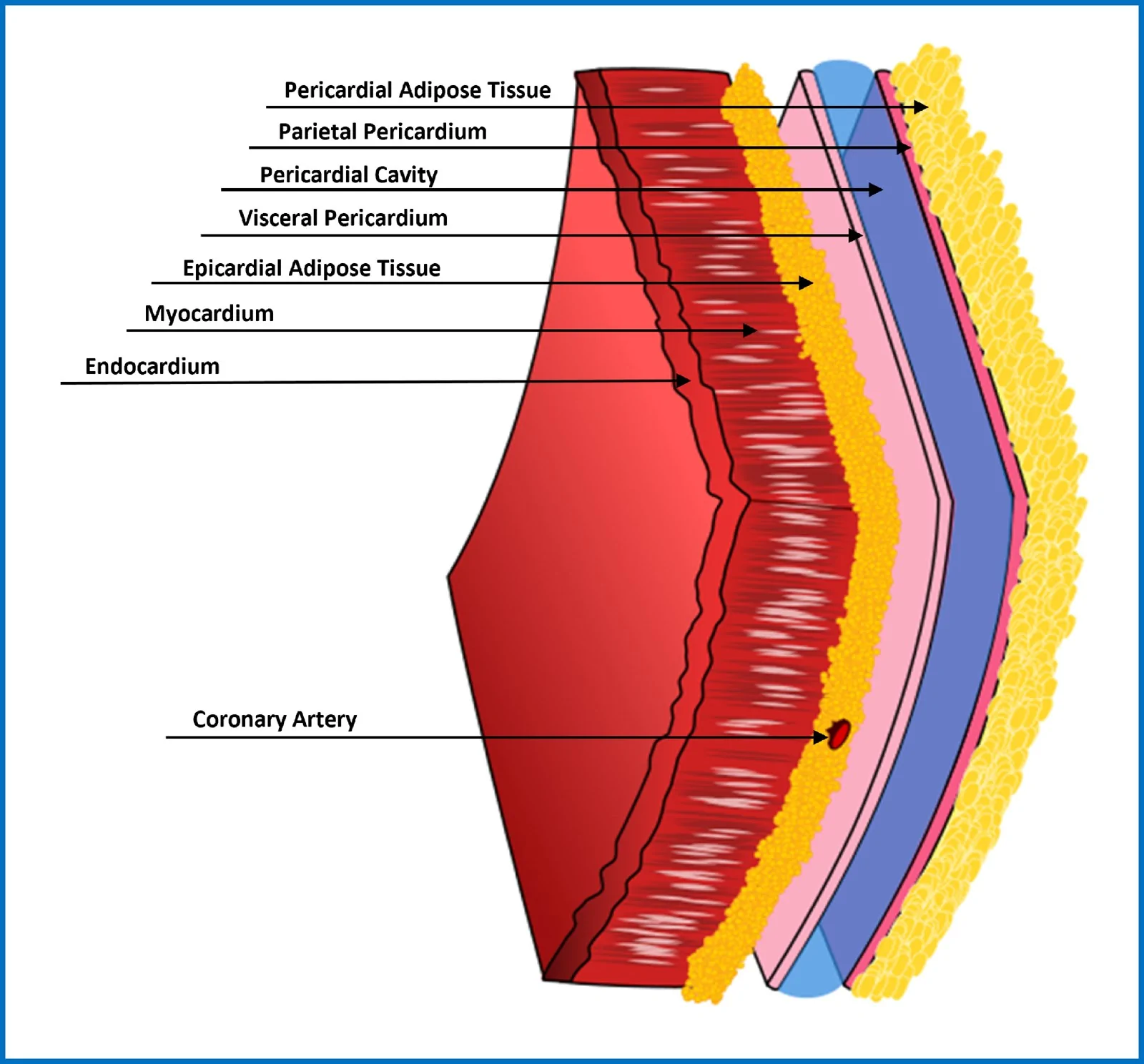
Schematic illustration of the different layers from inside (endocardium) to outside (PAT).

Precise definition and distinction of fat compartments has clinical relevance. Their distinct embryological origins, metabolic functions, and pathological implications contribute to diverse risk stratification and help understanding their specific roles in cardiovascular disease.^11^

EAT is classified as visceral fat, originates embryologically from the splanchnopleuric mesoderm and therefore has brown fat-like properties and functions.^12^ In adults, it is primarily located along the atrioventricular and interventricular grooves, with smaller deposits found along the atrial walls and around the atrial appendages. As EAT volume increases, it can extend between the ventricles and coronary arteries, even penetrating the myocardium due to the absence of a fascial barrier between EAT and the heart muscle. This unique anatomical relationship allows for direct metabolic and inflammatory interactions between EAT and the myocardium.^13^^,^^14^

In contrast, PAT originates from white adipose tissue and is metabolically more similar to subcutaneous fat.^15^ PAT is located outside the parietal pericardium, does not have direct contact with the myocardium, and surrounds the major, large blood vessels in the mediastinum. It is supplied by the pericardiophrenic artery, a branch of the internal thoracic artery, rather than from the coronary circulation. Thus, the effects of PAT on cardiovascular function are likely mediated through systemic rather than local mechanisms.

## Physiology

EAT exerts both paracrine and vasocrine effects on the myocardium and coronary arteries, influencing cardiovascular physiology through direct molecular signalling, while PAT is primarily involved in systemic metabolic regulation and inflammatory signalling.^16^ Expansion of both EAT and PAT has been increasingly linked to various cardiovascular diseases, including coronary artery disease (CAD), atrial fibrillation (AF), and heart failure (HF).

EAT is considered an active endocrine organ, secreting a range of bioactive molecules, including pro-inflammatory cytokines such as interleukin-6 (IL-6), tumour necrosis factor-alpha (TNF-α), and monocyte chemoattractant protein-1 (MCP-1). These adipokines contribute to endothelial dysfunction, oxidative stress, and vascular inflammation, thereby accelerating the progression of atherosclerosis.^17^ The close anatomical proximity of EAT to the coronary arteries suggests that these inflammatory mediators may exert direct paracrine effects on the vascular wall, promoting plaque development and instability.^17^ In addition to these pro-inflammatory effects, EAT also exerts protective functions under physiological conditions. It secretes beneficial adipokines such as adiponectin, which has anti-inflammatory and insulin-sensitizing properties, and syntheullin, a vasodilatory peptide with anti-ischemic effects. These mediators contribute to myocardial energy supply, vascular homeostasis, and endothelial protection. Thus, the function of EAT is context-dependent: while it plays a cardioprotective role in healthy individuals, its secretory profile can shift toward a pro-inflammatory phenotype under pathological conditions such as obesity, insulin resistance, or type 2 diabetes.^18^

In addition, EAT has been implicated in the pathogenesis of AF. Increased EAT volume has been associated with structural and electrical remodelling of the atria, likely due to the secretion of inflammatory and profibrotic cytokines. These mediators promote atrial fibrosis, altering conduction pathways and increasing susceptibility to arrhythmia. Furthermore, EAT may influence autonomic nervous system activity through sympathetic and parasympathetic modulation and contribute to AF development.^19^

EAT has also been linked to HF, particularly heart failure with preserved ejection fraction. The expansion of EAT may contribute to myocardial stiffness and diastolic dysfunction through the secretion of profibrotic factors such as transforming growth factor-beta (TGF-β) and leptin. These factors promote interstitial fibrosis, reducing ventricular compliance and impairing relaxation. Additionally, excessive EAT accumulation may exert mechanical compression on the myocardium, further worsening ventricular filling and cardiac workload.^20^

While less studied than EAT, PAT has been associated with systemic inflammation, insulin resistance, and endothelial dysfunction. Unlike EAT, which influences local myocardial and coronary function, PAT contributes to broader metabolic dysregulation. Increased PAT volume has been correlated with higher levels of circulating pro-inflammatory cytokines, which may exacerbate cardiovascular risk by promoting arterial stiffness and systemic inflammation. These findings suggest that both EAT and PAT play significant but distinct roles in cardiovascular disease progression.^21^^,^^22^

## Controversy

The definitive role and impact of EAT and PAT remains incompletely understood. Over the years, several publications have reported partially conflicting findings. For instance, Tanami et al^23^ found no significant association between EAT volume and severity of CAC, coronary stenosis, or myocardial perfusion abnormalities, raising questions about the clinical relevance of EAT quantification. Similarly, Mahabadi et al^24^ observed no significant correlation between high EAT volume and severe CAC score (≥400) in the general population, although a significant association was noted in patients below 55-years of age and with a low baseline CAC, suggesting potential age-dependent effects.

In contrast, multiple studies support the role of increased EAT volume or thickness as a predictor of adverse cardiovascular outcomes (Table 1). Chu et al,^40^ Tscharre et al,^41^ and Lu et al^42^ demonstrated that higher EAT thickness or volume significantly correlates with cardiovascular events in various patient cohorts, including those with AF, after acute coronary syndrome, or after percutaneous coronary intervention. Likewise, Eisenberg et al^43^ and West et al^44^ confirmed these associations, using deep learning-based methods for segmentation, further strengthening the case for EAT volume as a prognostic marker.

**Table 1.** EAT impact—a literature overview.

Author	Article type	Patient number	Follow-up^a^	Association between	Results	*P*-values	Segmentation method	Additional comments
**Imaging Modality: CT**								
**Fu Z et al. (2024)** ^25^	Meta-analysis	43 113	–	EAT & general abnormal fat distribution and HFpEF/diastolic dysfunction	Abnormal fat distribution was significantly greater in patients with cardiac diastolic dysfunction, especially in EAT.	**<.001**	Varied across included studies	Abnormal fat distribution was significantly correlated with the risk of developing cardiac diastolic dysfunction.
**Hendricks S et al. (2021)** ^26^	Meta-analysis	6600 (mainly CT)	-	EAT volume/thickness and myocardial infarction (MI)	Patients with myocardial infarction had 37% higher EAT measures compared to patients without MI. Echocardiography and CT showed similar relative differences in EAT.	**<.001**	Not reported	CT is considered gold standard; echocardiography is a practical alternative, especially in emergencies. More prospective studies needed for clinical routine use.
**Mancio J et al. (2018)** ^27^	Meta-analysis	41 534	–	EAT volume assessed by CT and CAD (including stenosis, calcification, ischemia, and MACE)	EAT volume is significantly higher in patients with CAD.	**<.001**	Not reported	Association is strongest for significant stenosis and MACE. Highlights EAT as a potential imaging biomarker in cardiovascular risk stratification.
**Monti CB et al. (2021)** ^28^ ^,^ ^29^	Meta-analysis	7683	-	EAT attenuation and CAD; EAT attenuation and segmentation thresholds	Patients with CAD have significantly higher EAT attenuation (−80.71 HU) compared to healthy individuals (−86.40 HU).	**.044**	Varied across included studies	EAT attenuation correlated to segmentation thresholds and EAT volume, highlighting variability in EAT attenuation due to technique; calls for standardization of HU thresholds
**Wong CX et al. (2016)** ^29^	Meta-analysis	352 275 (mainly CT)	–	EAT volume, abdominal adiposity, overall adiposity and AF	1- SD increase of EAT volume was associated with significantly higher odds of AF with stronger associations than abdominal or overall adiposity.	**<.001**	Varied across included studies	Epicardial fat showed stronger and more graded associations with AF than other adiposity measures. Suggests epicardial fat may be a more relevant and specific target for AF risk assessment.
**Imaging Modality: Echography**								
**Canpolat U et al. (2016)** ^30^	Clinical trial	234	1.5 years	EAT thickness and AF recurrence after cryoablation	Higher EAT thickness was an independent predictor of AF recurrence after cryoablation	** <.001**	Manual	EFT correlated with systemic inflammation marker (hs-CRP); study highlights importance of echocardiographic EFT in AF prognosis
**Majumder A R et al. (2022)** ^31^	Observational study	164	-	Echocardiographic EAT thickness and angiographic severity of CAD in acute coronary syndrome (ACS) patients	Higher EFT significantly associated with higher Gensini score (severity of CAD)	**<.001**	Manual	EAT thickness cutoff >4.65 mm predicted significant coronary stenosis with 76.1% sensitivity and 69.9% specificity; EFT may aid risk stratification
**Seo KW et al. (2024)** ^32^	Observational study	390	–	EAT thickness (Echo) and central fat distribution (truncal fat mass to total body fat mass ratio)	Females showed stronger correlation of EAT thickness with central fat than males	**.006** (males), **<.001** (females)	Manual	−EAT thickness measured on free wall of right ventricle; body fat distribution assessed by DXA; study highlights gender differences in fat distribution associations
**Yamaguchi S et al. (2020)** ^33^	Observational study	202	-	EAT thickness and LAA function (emptying flow velocity and orifice area)	EAT inversely correlated with LAA emptying flow	Flow *P* < .001; Area *P* = .014	Manual	Best cutoff for EAT thickness predicting low LAA emptying flow velocity was > 5.1 mm; study linked thick EAT with increased thromboembolic risk in AF
**Quisi A et al. (2018)** ^34^	Retrospective observational	216	–	EAT thickness and ECG markers (P-wave dispersion, corrected QT interval)	Higher EAT thickness linked with increased Pd and QTc	**.001** (Pd), **.004** (QTc)	Manual	EAT thickness independently associated with Pd, QTc, leukocyte count, and LV ejection fraction
**Imaging Modality: MRI**								
**Duca F et al. (2024)** ^35^	Observational study	966	4.8 years	EAT quality as a predictor of poor outcomes.	Higher EAT-T1 correlates independently with poor outcome.	**.026**	Manual	EAT quality but not EAT quantity, is independently associated with nonfatal myocardial infarction, heart failure hospitalization, and all-cause death.
**A. Dutour et al. (2016)** ^36^ ^,^ ^37^	Clinical trial	44	0.5 years	Exenatide treatment and changes in EAT & hepatic triglyceride content.	Exenatide led to: • ↓ EAT by −8.8% ± 2.1% • ↓ HTGC by −23.8% ± 9.5%	**.018** (EAT and weight loss)	Manual	Effects on EAT and HTGC not correlated with insulin resistance markers. Only weight loss significantly predicted fat reductions.
**Nakamori S et al. (2018)** ^37^	Observational study	105	–	Left atrial epicardial fat and presence of AF.	LA-EAT was significantly higher in AF patients (28.9 ± 12.3 mL) vs non-AF (14.2 ± 7.3 mL); remained significant after LA volume adjustment.	**<.001**	semi-automated segmentation	LA-EAT was independently associated with AF even after adjusting for LA volume and BSA. Combined LA-EAT + LA volume showed better prediction.
**Shao JW et al. (2024)** ^38^	Clinical trial	62	-	EAT/ LA LR diameter ratio and presence of HF with preserved ejection fraction in obese patients.	Obese patients with HFpEF showed significantly higher EAT volume, EAT mass, and EAT/left atrial diameter ratio compared to controls.	**<.05**	Manual	Authors suggest EAT/LA LR diameter ratio as a novel imaging biomarker. the EAT/LA diameter ratio was independently associated with HFpEF.
**Zhou Y et al. (2021)** ^39^	Observational study	93	–	EATVI and AF, along with LAVI and lower LVEF, in patients with HOCM.	Patients with HOCM and AF had significantly higher EATVI, LAVI, LVEF; EATVI was an independent predictor of AF, and combining EATVI, LAVI, and LVEF achieved excellent diagnostic performance.	**.023** (EATVI)	semi-automated segmentation	Demonstrates EATVI as an independent and strong imaging biomarker for AF in HOCM. The combination of EATVI, LAVI, and LVEF offers high diagnostic accuracy.

This table contains the 5 studies on EAT with the highest number of participants published in the past 10 years for CT, echocardiography, and MRI. Studies on PAT were not included due to the smaller number of studies and the smaller number of included participants. Boldface *P*-values indicate statistical significance (*P* < .05).

Abbreviations: ^a^Follow-up: Median in years, ACS = Acute Coronary Syndrome, AF = Atrial Fibrillation, AS CD = Aortic Stenosis and Cardiovascular Death, CAC = Coronary Artery Calcification, CAD = Coronary Artery Disease, CT = Computed Tomography, CACS = Coronary Artery Calcium Score, CV = Cardiovascular, DL = Deep Learning, DXA = Dual-energy X-ray Absorptiometry, EAT = Epicardial Adipose Tissue, EFV = Epicardial Fat Volume, ICA = Invasive Coronary Angiography, MACE = Major Adverse Cardiac Events, MP = Myocardial Perfusion, MRI = Magnetic Resonance Imaging, PCI = Percutaneous Coronary Intervention, SPECT = Single-Photon Emission Computed Tomography, TAVR = Transcatheter Aortic Valve Replacement.

Beyond quantity, recent studies have also focused on EAT quality. Pina et al^45^ found that both lower EAT volume and higher EAT attenuation were independently associated with increased all-cause mortality, while Duca et al^35^ highlighted that EAT tissue characteristics (eg, higher T1 times in MRI) were predictive of poor outcomes, even when EAT volume was not. These findings emphasize the growing recognition that not only the amount but also the composition and metabolic activity may play a role in disease progression.

Oikonomou et al^46^ introduced the fat attenuation index (FAI) as a potential surrogate marker for pericoronary inflammation, demonstrating that FAI independently predicts cardiovascular events—highlighting the prognostic value of EAT characteristics beyond volume alone. Huber et al showed that EAT dispersion, a CT-based measure of heterogeneity in fat distribution, is independently associated with AF recurrence after ablation^47^ and demonstrated that EAT characteristics (eg, texture and distribution) can further refine cardiovascular risk prediction.^48^ These findings highlight the increasing recognition of EAT’s functional and structural importance in risk stratification. However, controversy persists regarding the reproducibility of FAI across imaging platforms and populations, with ongoing investigations into standardizing CT scan, reconstruction, and measurement protocols.^49–51^

The causal mechanisms linking EAT volume and attenuation to cardiovascular outcome are not yet fully understood, but emerging data suggest a metabolic and inflammatory basis. Recent findings by Barchuk et al^52^ provide insight by demonstrating that EAT in CAD patients shows increased lipoprotein lipase (LPL) activity, which correlates with elevated levels of ceramides—bioactive lipids implicated in atherosclerosis. These ceramide species, particularly Cer18:1/24:1, were strongly associated with LPL activity and specific regulators such as GPIHBP1 and ANGPTL4. The study indicates that increased EAT volume may reflect metabolically active tissue rich in pro-inflammatory and atherogenic lipid species, while lower EAT attenuation (ie, higher lipid content) may mirror a detrimental tissue composition. This lipidomic profile may help explain the association between EAT characteristics and adverse cardiovascular outcomes, bridging the gap between imaging findings and pathophysiological relevance.^52^

## Imaging techniques

**Computed Tomography (CT)** is the most commonly used imaging modality for assessing EAT volume and attenuation due to its high spatial resolution, volumetric data, and the consistent and precise visualization of the pericardium. EAT can be measured on both non-contrast and contrast-enhanced CT images. The pericardium is contoured and voxels in a CT attenuation range between −190 and −30 Hounsfield Units (HU) are identified as adipose tissue.^53^ It is important to note that variations in acquisition parameters such as tube voltage (kVp) and tube current (mA) influence both attenuation and volume measurements of EAT when using fixed HU thresholds. In addition to CT acquisition parameters, other factors influence EAT attenuation as well. For example, seasonal variations have been shown to affect EAT attenuation, with lower HU values observed during summer compared to winter months. Furthermore, clinical and demographic variables—including sex, race, body mass index, diabetes status, and use of statins or antihypertensive medications—also have a relationship to EAT attenuation, potentially complicating its interpretation as a cardiovascular risk biomarker.^54^ Spearman et al suggested in their systematic review a volume threshold >125 mL as predictive of cardiac pathology.^55^

Importantly, non-contrast chest CT acquired using a tube voltage of 120 kVp and reconstructed with a slice thickness of 1.25 mm provides EAT volumes comparable to dedicated ECG-gated cardiac CT, offering broader applicability. Contrast-enhanced CT tends to underestimate EAT volume compared to non-enhanced scans, with a reported mean difference of 31 mL. Adjusting HU thresholds can improve consistency between protocols^56^^,^^57^ (Figure 2). A distinct advantage of CT for thoracic fat quantification is the possibility of quantifying adipose tissue in every chest CT scan, making it a potentially powerful tool for opportunistic screening.

**Figure 2.**
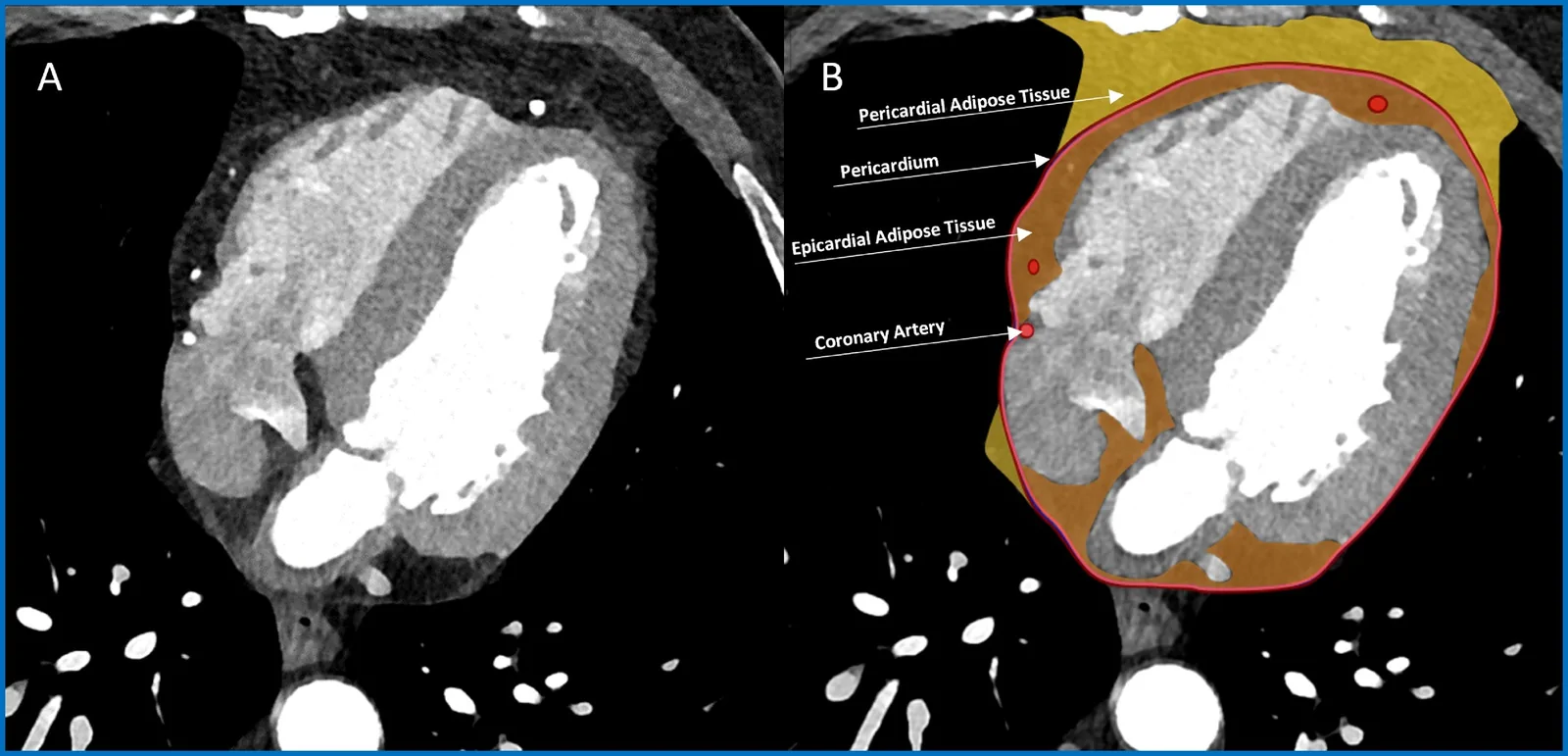
Cardiac CT of a 69-year-old male patient for evaluation of CAD. (A) Axial image at the level of the ventricles. (B) Schematic fat segmentation.

Beyond volume and attenuation metrics, radiomics-based approaches have enabled a more detailed tissue characterization of EAT.^58^ Zhang et al^59^ applied machine-learning algorithms to contrast-enhanced and non-enhanced chest CT to extract 1691 radiomics features from segmented EAT, including first-order statistics, shape descriptors, and texture-based features. Feature selection using the Boruta algorithm and classification via random forest models yielded an EAT-based radiomics signature (EAT-score), which demonstrated excellent performance in identifying AF (AUC 0.92 in contrast-enhanced and 0.85 in non-enhanced CT). The EAT-score significantly outperformed models based solely on EAT volume, attenuation, or clinical variables.

**Magnetic Resonance Imaging (MRI)** enables volumetric EAT quantification with high reproducibility and without radiation exposure (Figure 3). Various imaging sequences can be used, and administration of gadolinium-based contrast media is not required. This makes MRI particularly suitable for longitudinal studies. However, limitations include higher costs, longer acquisition times, and lower spatial resolution as compared to CT. Furthermore, larger slice thicknesses and sometimes limited pericardial definition, especially in inferior slices, can affect segmentation accuracy. Nevertheless, MRI remains a safe and reliable tool for EAT analysis.^60^^,^^61^

**Figure 3.**
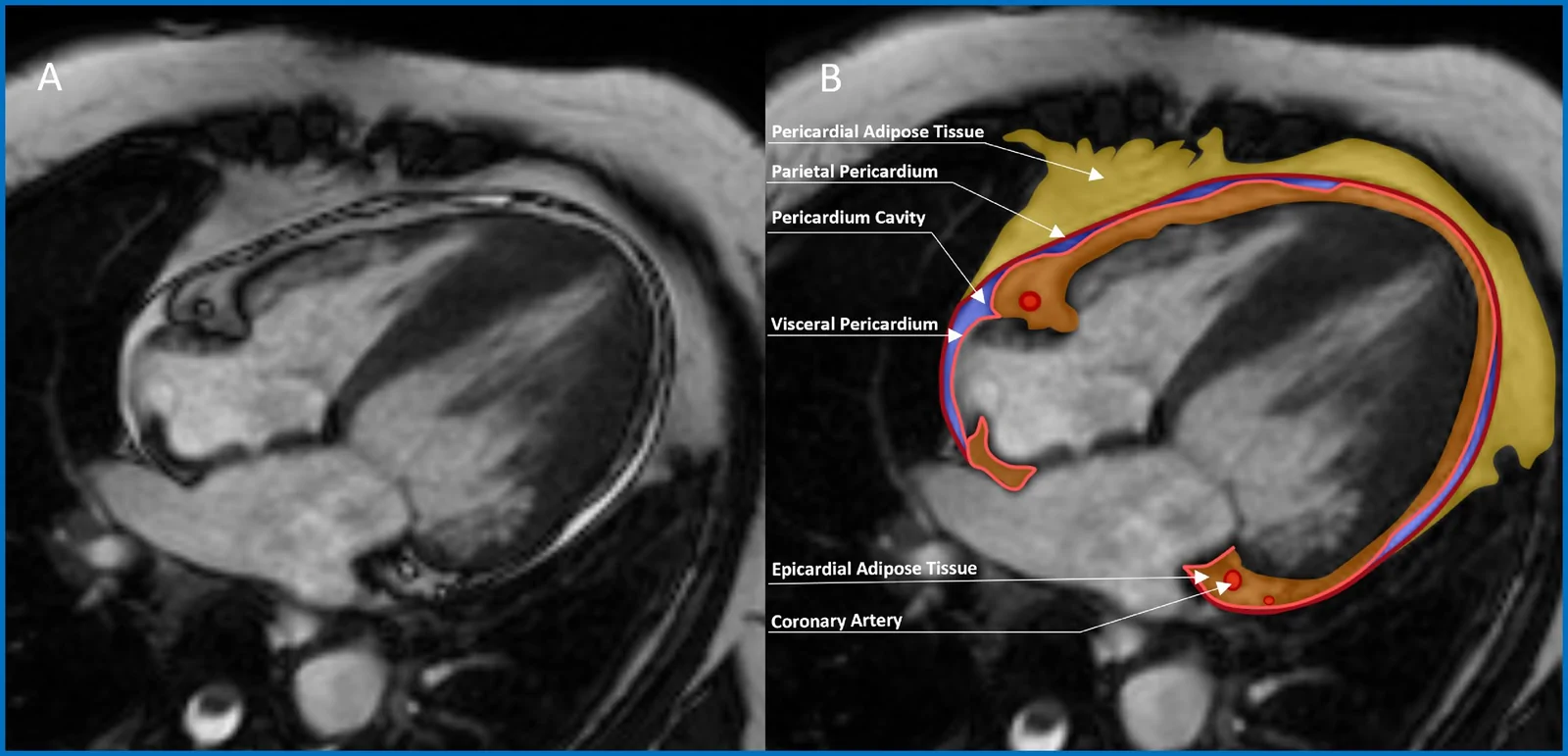
Cardiac MRI in a 54-year-old female patients with suspicion of myocardial fibrosis. (A) Axial image at the level of the ventricles. (B) Schematic fat segmentation.

Recent advances in cardiac MRI have significantly improved the accuracy and reproducibility of EAT assessment. Notably, Henningsson et al^62^ described a reduced intra- and inter-observer variability with cine 3D Dixon imaging compared to standard single-phase Dixon (ICC: 0.96 vs 0.92 and 0.76 vs 0.63, respectively), likely due to better delineation of epicardial borders across the cardiac cycle. The resulting time-resolved volumetric data enhances segmentation precision. Additionally, Dixon-based water-fat separation minimizes partial volume effects and improves tissue contrast for more accurate fat quantification.

Deep learning methods such as convolutional networks further automate EAT segmentation and achieve near expert-level performance (eg, Dice scores up to 0.77 in cine 4-chamber MRI). The recently developed EAT-Mamba pipeline, which integrates multiphase Dixon imaging with Res-Mamba blocks, has achieved Dice coefficients of 0.86 and highly accurate fat volume estimation.^63^

**Echocardiography** offers a non-invasive, low-cost, and radiation-free method to estimate EAT thickness, visualized as a hypoechoic space between the myocardium and the pericardium.^64^ In echocardiography, EAT is best detected over the right ventricular free wall, and thickness is measured perpendicularly in parasternal long- and short-axis views. A commonly used threshold of 5 mm has been proposed to identify individuals at increased cardiovascular risk.^65^ However, echocardiography does not allow volumetric assessment and is limited by low spatial resolution and high operator dependency. EAT may also be misinterpreted as pericardial effusion in some cases.^66^

Despite the individual advantages of each of the imaging techniques, there remains the challenge in achieving consistency and reproducibility across different studies and clinical settings, an issue which is particularly pronounced for echocardiography. Variability in imaging protocols, equipment calibration, and patient positioning can lead to differences in results, complicating the interpretation of findings. In CT imaging, several studies have demonstrated that variations in tube voltage and tube current influence both the attenuation and volume measurements of EAT, given that the HU threshold remains constant. Several studies have shown that variations in tube voltage influence EAT volume measurements when the HU-threshold remains constant. Reconstruction parameters, such as the use of iterative reconstruction techniques also influence HU-values and volume measurements, particularly when the predefined range of −190 to −30 HU is not being adapted.^51^^,^^67^

Many of the studies on the role of EAT and PAT on cardiovascular disease use variable imaging and measurement protocols, which hampers comparability. Therefore, standardization is crucial to ensure reproducibility, comparability, and clinical applicability.^68^^,^^69^

## Methods for adipose tissue segmentation

The development of medical image processing has undergone a significant transformation over the years. Initially, the segmentation of EAT and PAT was performed manually, requiring extensive effort and expert knowledge. This manual segmentation process was time-consuming, prone to errors, and subject to high inter-observer variability.^70^ In the following, techniques such as thresholding, region growing, and active contours dominated image segmentation. These methods relied on explicit rules and assumptions that were manually defined.^71^

To address these challenges, more advanced and automated methods have been introduced, with a growing emphasis on machine learning-based tools in recent years enabling automatic extraction of image features for segmentation. These approaches greatly improved the accuracy and flexibility of segmentation.^72–74^

In recent years, deep learning techniques have substantially enhanced the quality and efficiency of EAT and PAT segmentation. Convolutional neural networks, representing a distinct deep learning architecture, have proven effective for the automated segmentation of EAT and PAT. The primary advantages of these technologies lie in their ability to recognize complex patterns within image data and perform segmentation with minimal human intervention. This not only reduces time but also mitigates the inter-observer variability associated with manual and traditional semi-automated methods.^75^

Ding et al^76^ applied deep learning models to CT data for the automation of EAT segmentation, achieving high accuracy with a Dice coefficient of 0.82 compared to manual segmentation. Other studies, such as Rodrigues et al^77^ reported even higher performances, with deep learning models reaching Dice coefficients as high as 0.98. These results often exceed the variability encountered with manual segmentation, offering a promising solution for clinical applications.

An advantage of deep learning is its ability to learn from large datasets, enabling the model to handle various patient groups and image qualities. Through continuous learning and model refinement, the method can be further optimized. Once a model is trained and validated, results can be reliably reproduced on new datasets, improving the robustness and credibility of the method.^78^ Furthermore, deep learning has also been successfully used for predictive analysis: In a study on heavy smokers, EAT was used as a predictor of cardiovascular risk using neural networks.^79^

There are challenges associated with the application of deep learning in EAT and PAT segmentation. One of the primary obstacles is the need for large, annotated datasets for training, since the quality of deep learning models is directly dependent on the quantity and diversity of training data.^80^ In medical image processing, the acquisition of sufficient high-quality annotated image data is often difficult, which hinders the development and training of deep learning models. Solutions such as data augmentation techniques or synthetic image generation may help address the scarcity of training data.^80^

Moreover, deep learning models are often referred to as “black-box” models,^81^^,^^82^ meaning that their decision-making process can be challenging to interpret for human observers. In clinical practice, it is essential for the results produced by deep learning algorithms to be interpretable and validated. A lack of transparency in the model’s processes can thus present a barrier to widespread clinical adoption. Researchers are working on improving model explainability by incorporating techniques like Gradient-weighted Class Activation Mapping or Local Interpretable Model-agnostic Explanations, which aim to make the decision-making process of the models more understandable.^83^

Another challenge is the risk of overfitting, which may arise when models are trained on relatively small datasets. This may result in models that are not generalizable and may perform poorly on real-world clinical data. Consequently, it is crucial for deep learning models to be validated not only on specific datasets but also on diverse and broad patient populations to assess their true performance and susceptibility to errors.^80^

## Effects of treatment and lifestyle modification on EAT and PAT

The management of EAT accumulation, a key component of cardiometabolic risk, has attracted significant attention due to its potential role in cardiovascular diseases. Nonetheless, current evidence remains inconclusive as to whether EAT acts as an independent modifiable therapeutic target or serves as a surrogate marker of general visceral adiposity. Several therapeutic approaches including pharmacological interventions, lifestyle modifications, and surgical treatments, aim to reduce the volume of EAT and hence, the associated risks.^11^ Despite the fact that the relationship between adiposity and metabolic health is widely investigated, little is known about the treatment of pericardial fat.

Several cardiometabolic drugs have been investigated for their potential to reduce EAT volume, with effects observed for statins, GLP-1 receptor agonists (RA), and SGLT2 inhibitors.^84^ These drugs work through various mechanisms that contribute to reductions in EAT volume and thickness, and their impact has been assessed through a combination of imaging techniques such as ultrasound, CT, and MRI.^85^ In addition to volume reduction, anti-inflammatory effects have also been described. GLP-1 RA, such as Semaglutide showed a significant reduction in EAT inflammation and clinical improvement of psoriasis in obese patients with type 2 diabetes.^86^ EAT also expresses receptors for GIP, glucagon and GLP-1, which means that these drugs can potentially act directly on adipose tissue.^87^^,^^88^ GLP-1 receptor expression in the EAT was also associated with increased gene expression for fatty acid oxidation and white-to-brown fat transformation, suggesting possible metabolic reprogramming in the EAT by GLP-1 RAs.^89^

Statins are primarily known for their lipid-lowering effects, but recent studies suggest that they may also exert pleiotropic effects that contribute to EAT reduction. A meta-analysis of 3 studies based on ultrasound, CT or MRI involving 603 patients demonstrated that statins had a modest, yet statistically significant, impact on reducing EAT thickness measured in mm, ml or cm^3^ depending on the device. The standardized mean difference (SMD) for statins was −0.195 (95% CI −0.79, −0.32, *P* < .001), indicating a small reduction in EAT volume.^80^ Statins are thought to exert these effects not only through their lipid-lowering capabilities but also via their anti-inflammatory properties, which modulate adipose tissue function and metabolism. However, the overall impact of statins on EAT reduction remains relatively mild compared to newer agents like GLP-1 RA and SGLT2 inhibitors.^80^

Semaglutide has gained attention due to their effects on weight loss, glucose control, and cardiovascular protection. In a meta-analysis of 7 studies based on ultrasound, CT, and MRI involving 240 patients, GLP-1 RA demonstrated a substantial reduction in EAT thickness measured in mm, mL, or cm^3^ depending on the device. The SMD for GLP-1 RA was −1.005 (95% CI −1.37, −0.64, *P* < .001), indicating a robust effect on EAT reduction.^80^ Further sensitivity analyses confirmed that GLP-1 RA had a stronger effect on EAT reduction than SGLT2 inhibitors, with a 2-fold greater efficacy (*P* = .04). The proposed mechanisms for GLP-1 RA’s effect on EAT include induction of fat browning, improving myocardial insulin sensitivity, and possibly modulating inflammatory pathways. Furthermore, GLP-1 RA has been shown to have beneficial effects on the cardiovascular system, making it a promising candidate for managing EAT accumulation in patients with cardiometabolic risks.^85^

SGLT2 inhibitors, such as empagliflozin and canagliflozin, primarily target glucose metabolism and have demonstrated significant effects on reducing EAT. In 8 studies based on ultrasound, CT, or MRI involving 221 patients, SGLT2 inhibitors reduced EAT volume measured in mm, mL, or cm^3^ depending on the device with a SMD of −0.552 (95% CI −0.79, −0.32, *P* < .001). These drugs work by inhibiting the sodium-glucose cotransporter 2 in the kidneys, leading to increased glucose excretion and reduced blood glucose levels. The reduction in EAT with SGLT2 inhibitors is also thought to be mediated by improvements in body weight, inflammation, and fat oxidation. Interestingly, the effect of SGLT2 inhibitors on EAT was comparable to that of GLP-1 RA after 6 months, highlighting the potential of these drugs in the long-term management of EAT.^90^

However, recent findings from the cardiac MRI substudy of the SUMMIT trial challenge the assumption that EAT is consistently responsive to pharmacologic intervention. In that study, treatment with tirzepatide significantly reduced left ventricular (LV) mass and total paracardiac fat volume, but no specific effect on epicardial adipose tissue (EAT) was demonstrated, suggesting that observed reductions may reflect global fat loss rather than a direct modulation of EAT.^91^ These findings underscore the need for more precise phenotyping of cardiac adipose tissue compartments and support the notion that EAT may act as a secondary marker rather than a primary therapeutic target.

Lifestyle interventions, including dietary changes and increased physical activity, play a critical role in managing EAT and PAT in addition to pharmacological treatments. Both diet and physical exercise have been shown to significantly reduce EAT and PAT volume, though their effectiveness can vary depending on the approach.^92^ Particularly, the Mediterranean diet proved superior to others, such as the low-fat diet, in reducing EAT, likely due to its anti-inflammatory properties and high content of healthy fats.^93^ Aerobic exercise helps reduce visceral fat through enhanced fat oxidation and improved insulin sensitivity. Resistance training, while beneficial for increasing muscle mass, also aids in reducing fat depots such as EAT and PAT. High-intensity interval training has garnered attention for its efficiency in reducing visceral fat more effectively than traditional aerobic exercises.^94^

Multimodal therapy offers a comprehensive approach to manage EAT and reduce associated cardiovascular risks. GLP-1 receptor agonists, statins, and SGLT2 inhibitors contribute significantly to EAT reduction, with GLP-1 receptor agonists showing the most pronounced effect. Lifestyle changes further support these therapies, although bariatric surgery shows a significant reduction in EAT in meta-analyses, it remains a last line option for patients with severe obesity.^95^ Ongoing research is essential to refine these strategies and to enhance understanding of the mechanisms involved in EAT accumulation and its reduction.

PAT is typically reduced in parallel with EAT and other fat depots during weight loss, and although it may not be an independent therapeutic target, its regression may still contribute to improved cardiovascular outcomes. Ongoing research is needed to clarify the clinical relevance of PAT reduction and its potential mechanistic role in cardiometabolic disease.

## Future research directions

Future research should prioritize standardized imaging and measurement protocols to improve comparability and clinical applicability. A clearer distinction between EAT and PAT is needed, given their potentially different biological roles. Mechanistic studies are essential to determine whether EAT is a modifiable therapeutic target or merely a cardiovascular risk marker. Additionally, deep learning models should be further developed and validated to enable automated and interpretable analysis in clinical settings.

## Conclusion

This review aimed to summarize the growing recognition of EAT and PAT as key contributors to cardiovascular disease. The anatomical, physiological, and pathological roles of these tissues demonstrate their importance in cardiovascular risk stratification. Imaging techniques, particularly CT, have proven to be valuable tools for quantifying these fat depots, with deep learning-based segmentation methods offering further improvements in precision and reproducibility. Despite promising results, the exact role of EAT and PAT as independent cardiovascular risk factors remains controversial, with conflicting findings across various studies. Nonetheless, the current belief is that both EAT and PAT significantly impact cardiovascular health, with EAT emerging as a predictor of adverse outcomes.

Anatomical precision in imaging and segmentation is essential. In this context, the continued development and standardization of imaging protocols, alongside the integration of advanced imaging techniques and machine learning tools, will be pivotal in translating these findings into clinical practice.
